# Application of Nanoscopic Quantum Systems in Retinal Restoration

**DOI:** 10.18502/jovr.v19i1.15415

**Published:** 2024-03-14

**Authors:** Hadi Mohammadi, Hashem Rafii-Tabar

**Affiliations:** ^1^Department of Medical Physics and Biomedical Engineering, School of Medicine, Shahid Beheshti University of Medical Sciences, Tehran, Iran; ^2^Nanomedicine Research Association (NRA), Universal Scientific Education and Research Network (USERN), Tehran, Iran; ^3^The Physics Branch of the Academy of Sciences of Iran, Tehran, Iran

Ophthalmic diseases pose a significant challenge to the well-being and quality of life of millions of individuals globally, as they could cause vision loss in severe cases. One such condition is the Retinitis Pigmentosa (RP) group of rare eye diseases that affect the retina. This is an inherited degenerative disorder indicative of the gradual deterioration of rod and cone photoreceptors^[1]^. Furthermore, age-related macular degeneration (AMD), a disease affecting the macula – the central region of the retina responsible for sharp vision – presents another example of a condition with serious implications for the vision health. The AMD is the leading cause of blindness among individuals aged 55 and older^[2,3]^, affecting approximately 67 million people in the EU^[4]^ and 11 million in the US^[3]^, with a global affliction of 170 million. Prognosis suggest that the prevalence of the AMD in the US will rise to 22 million by 2050, and globally to 288 million by 2040^[2,3]^. In this short note we briefly report on new attempts by scientists and clinicians to confront these kinds of diseases by using nanoscopic quantum systems to cope with such neuro degenerative disorders.

To date, various traditional methods have been employed for the treatment of retinal degenerative diseases, including both noninvasive and invasive approaches. For example, noninvasive treatment of the RP and AMD, such as pharmacological and neuroprotection agents therapy^[5]^, show promise for the early-stage intervention, whereas more invasive therapies, including photodynamic laser treatment^[6]^ and anti-VEGF injections^[7]^ become necessary for more advanced stages of the disease. Notwithstanding these therapies, the challenge still remains to find a more effective solution for the treatment of the retinal degenerative diseases.

In recent years, an alternative approach to the conventional treatment of the RP and AMD has emerged through the application of the extracellular electrical stimulation technique of both the inner and/or outer retinal network, leading to the development of biocompatible implantable engineered electronic prostheses, such as an array of electrodes that convert optical signals into electrical signals, which are then transmitted to the brain for neural processing and image formation. Furthermore, prostheses, such as the sub- and epi-retinal ones, could be implanted at different locations within the retina. Recently, some electronic-based prostheses, such as Argus II^[8]^ and Alpha IMS^[9]^, have received human clinical trial approval. Although retinal prostheses have demonstrated successful stimulation of retinal networks, nevertheless technical challenges such as the need for a complicated surgery, post-surgery complications and undesired side effects, low spatial and temporal regulations^[10,11,12,13]^ have significantly limited their use in human applications thus far.

Recent advancements in regenerative medicine, such as nanomedicine and/or medical nanotechnology, have opened new vistas as promising fields in attempts to develop innovative treatments to restore vision in individuals with retinal degenerative and optic nerve diseases^[14,15,16]^.These new fields involve the use of nanoscale materials and devices to target and interact locally with specific cells and tissues in the eye^[17,18]^. One application is the use of nanocarriers for targeted drug^[19]^ and gene^[20]^ delivery to the retina^[21]^, allowing for a precise and controlled release of therapeutic agents, such as nanoparticle-based vehicles for gene delivery to inherited retinal diseases using CRISPR-Cas9^[20]^. Additionally, nanoscopic devices enable further improvement of the current implants through amplification of their physio-chemical properties, providing enhanced functionality and biocompatibility. In this regard, bare carbon nanotubes (CNTs)^[22]^ or dressed CNTs^[23]^ have been used to modify both the electrical and mechanical features of microelectrode chips via a coating process at the interface with the retinal neurons. Other applications of nanomedicine relate to nanoscale implants and devices that can interface with the retinal cells^[23,24,25,26,27,28]^, such as TiO ${\;}_{2}$ -Au nanowires that could be implanted directly, acting as photoreceptor cells for transduction of the light signal into the electrical signal^[28]^.

A recent development has been the use of quantum systems in retinal restoration, namely the intravital injection of quantum dots (QDs) for restoring the visual function in human damaged retina^[29]^. These exotic nanoscopic semiconductor particles, which are implanted via intravitreal injection, possess unique optical and electrical properties that make them suitable for specific applications in the retinal tissue^[30]^. Preclinical studies have evaluated the efficacy of silicon-cadmium selenide (CdSe) QDs for preventing the progressive damage of the photo-sensitive cells through intraocular injection^[31,32]^. The results show that the intravitreal injection of silicon QDs is safe and does not show any sign of toxic or allergic reactions. Moreover, the electrical activity of the retina after the injection of photo-active QDs is increased significantly. These advances in preclinical investigations hold great potential and offer new hope for patients affected by the RP. This approach was investigated, in the first human safety and efficacy clinical study, on 20 subjects in two groups; group A (5 adults with end stage) group B (15 with severe RP), with two dosages of cadmium/selenium 655 Alt QDs, including 0.2 or 2 M, injected intravitreally to both groups^[29]^.

In conclusion, the integration of nanoscopic systems and ophthalmology in search to find an effective approach or tool for restoring vision has emerged as a novel innovation with the potential to significantly impact ophthalmology and vision science. The aforementioned approaches have shown that the use of nanoscale systems to help restore vision and create a protective effect on nerve cells has been successful. Indeed, recent advancements in this field represent a paradigm shift in the approach to the treatment of the retinal degenerative diseases such as the AMD and RP. The potential of nanoscience along with nanotechnology to revolutionize targeted drug/electrical field/gene delivery, optogenetic therapy, and enhanced CRISPR/CAS9 technique, offer new hope for patients and clinicians alike. While challenges persist, the pursuit of innovative, multidisciplinary approaches (basic science, clinical) nanomedicine-based solutions in ophthalmology has created optimism for a future where vision loss may be effectively mitigated or even reversed.

##  Financial Support and Sponsorship

None.

##  Conflicts of Interest

None.

## References

[B1] Wang AL, Knight DK, Thanh-thao TV, Mehta MCJIoc (2019). Retinitis pigmentosa: Review of current treatment. Int Opthalmol Clin.

[B2] García-Layana A, Cabrera-López F, García-Arumí J, Arias-Barquet L, Ruiz-Moreno JMJCiia (2017). Early and intermediate age-related macular degeneration: Update and clinical review. Clin Interv Aging.

[B3] Pennington KL, DeAngelis MM

[B4] Li JQ, Welchowski T, Schmid M, Mauschitz MM, Holz FG, Finger RPJBJoO (2020). Prevalence and incidence of age-related macular degeneration in Europe: A systematic review and meta-analysis. Br J Ophthalmol.

[B5] Mata NL, Vogel RJCoio (2010). Pharmacologic treatment of atrophic age-related macular degeneration. Curr Opin Ophthalmol.

[B6] Schmidt-Erfurth U, Hasan TJSoo (2000). Mechanisms of action of photodynamic therapy with verteporfin for the treatment of age-related macular degeneration. Surv Ophthalmol.

[B7] Holz FG, Schmitz-Valckenberg S, Fleckenstein MJTJoci (2014). Recent developments in the treatment of age-related macular degeneration. J Clin Invest.

[B8] da Cruz L, Dorn JD, Humayun MS, Dagnelie G, Handa J, Barale P-O, et al (2016). Five-year safety and performance results from the Argus II retinal prosthesis system clinical trial. Ophthalmology.

[B9] Stingl K, Bartz-Schmidt KU, Besch D, Chee CK, Cottriall CL, Gekeler F, et al (2015). Subretinal visual implant alpha IMS – Clinical trial interim report. Vision Res.

[B10] Winter JO, Cogan SF, Rizzo J (2007). Retinal prostheses: Current challenges and future outlook. J Biomater Sci Polym Ed.

[B11] Picaud S, Sahel J-A (2014). Retinal prostheses: Clinical results and future challenges. C R Biol.

[B12] Schmid EW, Fink W, Wilke R (2014). Operational challenges of retinal prostheses. Med Eng Phys.

[B13] Zeng Q, Huang Z (2023). Challenges and opportunities of implantable neural interfaces: From material, electrochemical and biological perspectives. Adv Funct Mater.

[B14] Zarbin MA, Montemagno C, Leary JF, Ritch R (2013). Nanomedicine for the treatment of retinal and optic nerve diseases. Curr Opin Pharmacol.

[B15] Zarbin MA, Arlow T, Ritch R (2013). Regenerative nanomedicine for vision restoration. Mayo Clin Proc.

[B16] Gómez-Garzón M, Martínez-Ceballos MA, Gómez-López A, Rojas-Villarraga A

[B17] Zarbin MA, Montemagno C, Leary JF, Ritch R (2010). Nanomedicine in ophthalmology: The new frontier. Am J Ophthalmol.

[B18] Tang Z, Fan X, Chen Y, Gu P (2022). Ocular nanomedicine. Adv Sci.

[B19] Herrera-Barrera M, Ryals RC, Gautam M, Jozic A, Landry M, Korzun T, et al (2023). Peptide-guided lipid nanoparticles deliver mRNA to the neural retina of rodents and nonhuman primates. Sci Adv.

[B20] Chien Y, Hsiao Y-J, Chou S-J, Lin T-Y, Yarmishyn AA, Lai W-Y, et al (2022). Nanoparticles-mediated CRISPRCas9 gene therapy in inherited retinal diseases: Applications, challenges, and emerging opportunities. J Nanobiotechnology.

[B21] Tawfik M, Chen F, Goldberg JL, Sabel BA, et al (2022). Nanomedicine and drug delivery to the retina: Current status and implications for gene therapy. Naunyn Schmiedebergs Arch Pharmacol.

[B22] Eleftheriou CG, Zimmermann JB, Kjeldsen HD, David-Pur M, Hanein Y, Sernagor E (2017). Carbon nanotube electrodes for retinal implants: A study of structural and functional integration over time. Biomaterials.

[B23] Bareket L, Waiskopf N, Rand D, Lubin G, David-Pur M, Ben- Dov J, et al (2014). Semiconductor nanorod–carbon nanotube biomimetic films for wire-free photostimulation of blind retinas. Nano Lett.

[B24] Riva  ER, Micera S

[B25] Zhao D, Huang R, Gan J-M, Shen Q-D (2022). Photoactive nanomaterials for wireless neural biomimetics, stimulation, and regeneration. ACS Nano.

[B26] Choi C, Choi MK, Liu S, Kim M, Park OK, Im C, et al (2017). Human eye-inspired soft optoelectronic device using high-density MoS2-graphene curved image sensor array. Nat Comm.

[B27] Ghaffari M, Moztarzadeh S, Rahmanian F, Yazdanpanah A, Ramedani A, Mills DK, et al (2016). Nanobiomaterials for bionic eye: Vision of the future. Eng Nanobiomater.

[B28] Tang J, Qin N, Chong Y, Diao Y, Wang Z, et al (2018). Nanowire arrays restore vision in blind mice. Nat Commun.

[B29] Jackson TL, Mandava N, Quiroz-Mercado H, Benage M, Garcia-Aguirre G, Morales-Canton V, et al (2021). Intravitreal quantum dots for retinitis pigmentosa: A first-in-human safety study. Nanomedicine.

[B30] Gao X, Cui Y, Levenson RM, Chung LWK, Nie S (2004). In vivo cancer targeting and imaging with semiconductor quantum dots. Nat Biotechnol.

[B31] Olson JL, Velez-Montoya R, Mandava N, Stoldt CR (2012). Intravitreal silicon-based quantum dots as neuroprotective factors in a model of retinal photoreceptor degeneration. Invest Ophthalmol Vis Sci.

[B32] Olson J, Velez-Montoya R, Mandava N, Stoldt CR (2013). Neuroprotective effect of photoactive quantum dots in progressive retinal photoreceptor degeneration. J Nanomater Mol Nanotechnol.

